# Can Low Cortisol Predict Long COVID? A Controversial Issue

**DOI:** 10.3390/biomedicines13112636

**Published:** 2025-10-27

**Authors:** Tom C. Quach, Alina Wilson, Oliver Sum-Ping, Sara Lomba, Linda N. Geng, Robert Shafer, Mitchell G. Miglis, Phillip C. Yang, Lauren Grossman, Giorgio Ricciardiello, Hector Bonilla

**Affiliations:** 1 Stanford University School of Medicine, Stanford, CA 94305, USA; 2 Division of Sleep Medicine, Stanford Medicine, Stanford, CA 94305, USA; 3 Department of Medicine, Stanford Medicine, Stanford, CA 94305, USA; 4 Division of Infectious Diseases, Stanford Medicine, Stanford, CA 94305, USA; 5 Department of Neurology and Neurological Sciences, Stanford Medicine, Stanford, CA 94305, USA; 6 Department of Cardiovascular Medicine, Stanford Medicine, Stanford, CA 94305, USA; 7 Department of Psychiatry and Behavioral Sciences, Stanford Medicine, Stanford, CA 94305, USA

**Keywords:** long COVID, post-acute COVID-19 syndrome, cortisol, circadian rhythm, long COVID biomarkers, hypocortisolism, hypercortisolism

## Abstract

Cortisol dysregulation has been proposed as a biomarker of long COVID (LC), but findings remain inconsistent. Prior reports suggested low cortisol levels in LC, yet collection times and study designs varied substantially. To evaluate morning serum cortisol distributions in an independent LC cohort, accounting for circadian timing and sleep patterns, we performed a retrospective cross-sectional study of consecutive adults seen at the Stanford Long COVID Clinic between 14 February 2022 and 31 July 2024 (IRB #62996). Eligible participants had confirmed SARS-CoV-2 infection, symptoms persisting ≥3 months per NASEM criteria, completion of the Alliance Sleep Questionnaire (ASQ), and a morning serum cortisol measured using the Roche Elecsys^®^ Cortisol II assay. Analyses were restricted to collections between 05:00–10:00, categorized as early morning peak (EMP: 05:00–08:00) or mid-morning (MMP: 08:01–10:00). Cortisol was classified as low (<6.2 μg/dL), normal (6.2–19.4 μg/dL), or elevated (>19.4 μg/dL). Among 86 LC patients (69.8% female; mean age 45.4 ± 12.9 years), the mean serum cortisol level was 15.67 ± 6.76 μg/dL. Overall, 62.8% of patients had cortisol within the reference range, 36.0% had elevated levels, and only 1.2% (*n* = 1) had a low value. Cortisol distributions were comparable across the EMP and MMP collection windows, with no statistically significant differences observed by sleep alignment. Inflammatory markers, including CRP and D-dimer, were largely within reference ranges across all cortisol strata. Contrary to earlier reports, low morning cortisol was rare in this LC cohort; most values were normal or elevated. Findings underscore the importance of circadian timing when interpreting cortisol in LC and highlight the need for prospective studies with serial measurements to determine biomarker utility.

## 1. Introduction

Immune dysregulation is recognized as a contributor to long COVID (LC) and its symptoms impacting various bodily systems [1]. With a lack of LC biomarkers, diagnosis is based on the history and documentation of SARS-CoV-2 infection and clinical symptoms. The most current case definition of LC was set by the National Academies of Science, Engineering, and Medicine (NASEM) [2], at three months or longer post-COVID-19 infection. Cortisol, a glucocorticoid hormone produced by the adrenal glands, has been found to be a significant predictor of LC status at lower levels, related to the roles in inflammation and disease pathobiology in a past landmark study [3]. Given our clinical observation of normal cortisol within Stanford LC patients and recent studies [4] questioning the reliability of observed low cortisol–LC relationships, we assessed our independent LC cohort to provide updated evidence of cortisol levels and the impact from sleep patterns.

## 2. Methods

We conducted a retrospective cross-sectional study of consecutive patients evaluated at the Stanford Long COVID (LC) Clinic between 14 February 2022 and 31 July 2024, under Stanford IRB approval #62996. Eligible participants had documented prior SARS-CoV-2 infection, symptoms consistent with Long COVID for ≥3 months per the NASEM case definition, completion of the Alliance Sleep Questionnaire (ASQ), and a same-encounter morning serum cortisol measurement. Exclusions were missing the ASQ or absence of morning cortisol. Serum cortisol (μg/dL) was assayed using Roche Elecsys^®^ Cortisol II (monoclonal antibody) (Indianapolis, IN, USA); morning reference range was defined between 6.2–19.4 μg/dL with defined categories as low (<6.2), normal (6.2–19.4), and elevated (>19.4). To address circadian variation, we restricted analyses to specimens collected from 05:00–10:00 and classified sampling times a priori as Early Morning Peak (EMP: 05:00–08:00) or Mid-Morning (MMP: 08:01–10:00); no samples collected after 10:00 were included. In clinical laboratories, morning cortisol is considered to be samples collected between 5:00 and 10:30 [5]. Subsequent determination of alignment between ASQ responses and timepoint of cortisol measurement was decided based on the subjective circadian measure recorded from patient ASQ response.

Covariates included demographics (age, sex, race), body mass index (BMI), comorbidities, LC symptom duration, inflammatory markers (CRP, D-dimer), and corticosteroid exposure abstracted from the electronic health record, as well as sleep symptoms and severity derived from the ASQ. Continuous variables were summarized as mean ± SD, and categorical variables as n (%). Between-group comparisons of continuous measures used *t*-tests or ANOVA for normally distributed data and Mann–Whitney *U* or Kruskal–Wallis tests otherwise; categorical comparisons used χ^2^ or Fisher’s exact tests. Cortisol distributions across circadian windows and alignment categories were compared using both parametric and nonparametric tests, and results were visualized with boxplots to evaluate diurnal patterns.

Multivariate ordinal logistic for cortisol level at 19.4 µg/dL binary cut off was fitted to evaluate associations between clinical, sleep, and demographic variables and cortisol levels. Models included robust (HC3) standard errors and were adjusted for age, sex, BMI, race, vaccination status, and circadian collection phase. Odds ratios with 95% confidence intervals and p-values were reported. All analyses were two-sided with α = 0.05, performed in Python 3.9 (SciPy v1.12.0, Statsmodels v0.14.0, Seaborn v0.12.2).

## 3. Results

Among our cohort (*n* = 86), a majority were female (69.8%) and white (72.1%), with an average age of 45.44 (SD 12.90) years and BMI of 26.86 (SD 6.17). A total of 54 (62.8%), 31 (36.0%), and 1 (1.16%) patient(s) had normal, elevated, and low levels, respectively (Table 1). Overall median cortisol was 15.67 µg/dL (SD 6.76 µg/dL), with a minimum of 5 µg/dL and a maximum of 38.1 µg/dL. Median symptom duration was 799 days (SD 383.37 days). C-Reactive Protein (CRP) and d-dimer levels were normal in 84.3% (43/51), 80.0% (20/25), and 100% (1/1) of the patients with normal, high, and low cortisol cohorts, respectively. D-dimer levels were within normal limits for 83.3% (25/30), 88.9% (16/18), and 100% (1/1), respectively. Additional characteristics are presented in Table 1 and Table S1.

Comparison of alignment versus non-alignment among the EMP and MMP cohorts revealed no statistically significant differences. All groups, EMP Aligned (*n* = 5), EMP Non-Aligned (*n* = 11), MMP Aligned (*n* = 41), and MMP Non-Aligned (*n* = 29), were concentrated in the normal cortisol range. Mean (SD) levels were 16.86 (4.20), 15.15 (5.37), 14.59 (6.00), and 15.86 (7.26) µg/dL, respectively (Supplementary Materials Figure S1).

In multivariate logistic regression adjusted for age, sex, BMI, race, vaccination status, functional stage, and sleep characteristics, no variable showed a statistically significant association with elevated morning cortisol (all *p* > 0.05). Odds ratios with 95% confidence intervals are shown in Table 2.

## 4. Discussion

A central circadian clock regulates cortisol levels within the hypothalamus’s suprachiasmatic nucleus. The awakening response increases cortisol within the first hour after awakening, with a daily peak around the habitual sleep–wake transition and minimal levels in the evening and early night [6,7]. 98.84% of our LC participants had normal (62.79%) or elevated (36.05%) cortisol. The single low cortisol patient had a concomitant Adrenocorticotropic Hormone (ACTH) value of 22 pg/mL (normal range: 7.2–63.3 pg/mL), without prior medical and corticosteroid histories. Thus, our cohort results suggest that low cortisol may not define LC, a condition where patients may experience chronic systemic inflammation for multiple years. The timing of blood collection can explain these discrepancies between our study and past studies that located significant relationships between low cortisol levels and long COVID [3,8]. Our plasma cortisol levels were collected between 5:00 a.m. and 10:00 a.m., well within typical cortisol peak windows. Normal and elevated cortisol did not significantly correlate with specific LC characteristics or symptoms [9].

We were concerned that the concomitant use of steroids may interfere with the cortisol measurement. The cortisol plasma concentration level was determined by using the Roche Elecsys immunoassay. A cross-reactivity of 5% or greater on this assay was found with 6β-hydroxycortisol, allotetrahydrocortisol, 21-deoxycortisol, fludrocortisone, prednisolone, and 6-methylprednisolone. We had patients who were on systemic steroids, 9 (10.74%) for budesonide, 5 (5.81%) for dexamethasone, 2 (6.45%) for hydrocortisone, 2 (2.33%) for prednisone, and 3 (3.49%) on topical/local steroids (triamcinolone). Prednisolone and 6-methylprednisolone can contribute to a substantially high cortisol concentration [10]. Overall, the small number of this cohort’s patients on steroids that affected cortisol levels did not impact the study’s results.

The general absence of hypercortisolism and hypocortisolism in these EMP and MMP circadian rhythm alignment sub-groups suggests that cortisol secretion is not severely dysregulated in LC-induced sleep disorders and may be less sensitive to circadian misalignments [11]. No correlations were found between cortisol levels and time of awakening.

Our investigation contains multiple strengths, including a defined morning collection timeframe, a clinically diagnosed LC cohort and standardized cortisol detection assays. Limitations include the retrospective nature and response limitations from surveys. The time of collection since awakening, comorbidities, and ongoing corticosteroid use may have also affected cortisol levels [12,13]. It is important to consider that cortisol is a variable hormone that presents with circadian fluctuation throughout the day. Thus, cortisol measurements collected at a single time point for each patient may not be sufficient in establishing a comprehensive picture of cortisol’s relationship with the LC condition. Unmeasured confounding, such as the exact time since awakening, may have influenced cortisol variability. Future prospective studies with standardized awakening assessments are warranted and subjective responses should be collected with a shorter temporal window. The smaller sample size of the Stanford LC cohort, a single academic medical center location, may limit its generalizability and possess a reduced statistical power for definitive findings, as opposed to larger-scale, multi-site studies. Further prospective investigations with longitudinal tracking of cortisol trends in LC patients are needed to elucidate the full scope of endocrine and immune biomarkers in this population.

## Figures and Tables

**Table 1:** Demographic and clinical characteristics of the Stanford Long COVID (LC) cortisol cohort (*n* = 86). Patients are stratified by morning cortisol category—low (<6.2 μg/dL), normal (6.2–19.4 μg/dL), and elevated (>19.4 μg/dL)—based on Roche Elecsys^®^ Cortisol II reference ranges. Values are presented as mean ± SD for continuous variables and n (%) for categorical variables.

Variable	Normal	High	Low	Total (n)	*p*-Value
**Count**	54 (62.79%)	31 (36.05%)	1 (1.16%)	86 (100%)	
**Cortisol Level (µg/dL)** Median (SD)	11.7 (3.14)	23.02 (4.72)	5.0 (-)	86 (100%)	
**Adrenocorticotropic Hormone (ACTH)** Median (SD)	24.37 (13.44)	28.07 (24.16)	22.0 (-)	39 (45.35%)	
**Age** Median (SD)	47.31 (11.16)	42.23 (15.79)	44.0 (-)	86 (100%)	0.1801
**BMI** Median (SD)	27.46 (5.44)	25.62 (6.7)	32.92 (-)	86 (100%)	0.055
**Race**				86 (100%)	
White/Caucasian	36 (66.67%)	23 (74.19%)	0 (0%)	59 (68.6%)	
Black/African American	3 (5.56%)	0 (0.0%)	0 (0%)	3 (3.49%)	
Native American/Alaska Native	1 (1.85%)	1 (3.23%)	0 (0%)	2 (2.33%)	
Asian	8 (14.81%)	3 (9.68%)	1 (100.0%)	12 (13.95%)	
Multi-racial	6 (11.11%)	4 (12.9%)	0 (0%)	10 (11.63%)	
**Sex At Birth**				86 (100%)	0.8135
Male	16 (29.63%)	10 (32.26%)	0 (0%)	26 (30.23%)	
Female	38 (70.37%)	21 (67.74%)	1 (100%)	60 (69.77%)	
**Comorbidities**					
Diabetes	1 (1.85%)	4 (12.9%)	0	5 (5.81%)	
Hypertension	9 (16.67%)	5 (16.13%)	0	14 (16.28%)	
**Prior use of corticoids**	24 (44.44%)	12 (38.71%)	0	36 (41.86%)	
**Current use of corticoids**	23 (42.59%)	14 (45.16%)	0	37 (43.02%)	
Budesonide	7 (12.96%)	2 (6.45%)	0	9 (10.47%)	
Dexamethasone	3 (5.56%)	2 (6.45%)	0	5 (5.81%)	
Hydrocortisone	0 (0.0%)	2 (6.45%)	0	2(2.33%)	
Prednisone	2 (3.7%)	0 (0.0%)	0	2 (2.33%)	
Triamcinolone	2 (3.7%)	1 (3.23%)	0	3 (3.49%)	
**Low-Dose Naltrexone**	15 (27.78%)	12 (38.71%)	0 (0.0%)	27 (31.4%)	
**Functional Status**				85	0.5902
2—No limitations but I felt the symptoms	11 (20.37%)	3 (9.68%)	0 (0%)	14 (16.47%)	
3—I avoided some of my daily activities	22 (40.74%)	15 (48.39%)	1 (100%)	38 (44.71%)	
4—I struggled to take care of myself	14 (25.93%)	12 (38.71%)	0 (0%)	26 (30.59%)	
5—I was in bed all the time	6 (11.11%)	1 (3.23%)	0 (0%)	7 (8.24%)	

**Table 2:** Multivariate logistic regression assessing predictors of elevated morning cortisol among Long COVID patients. No variable demonstrated a statistically significant association with elevated morning cortisol. Odds ratios (OR) and corresponding 95% confidence intervals (CI) are provided.

Variable	OR (95% CI)	*p*-Value
Age	0.99 (0.61–1.59)	0.958
Gender	1.03 (0.37–2.91)	0.950
BMI	0.59 (0.29–1.20)	0.145
Race	0.82 (0.60–1.13)	0.225
COVID vaccination status	0.99 (0.21–4.66)	0.994
Days ASQ Cortisol	0.64 (0.37–1.11)	0.112
Functional Stage	1.07 (0.65–1.76)	0.780
Change in smell	0.79 (0.40–1.56)	0.496
Change in taste	0.73 (0.38–1.39)	0.337
Breathing symptoms	1.38 (0.54–3.58)	0.502
rMEQ	1.25 (0.76–2.05)	0.386

## Data Availability

Data available upon request, due to medical privacy surrounding personal health information.

## Supplementary Materials

The following supporting information can be downloaded at: https://www.mdpi.com/article/10.3390/biomedicines13112636/s1, Figure S1: Circadian and collection time alignment and non-alignment among the EMP and MMP cohorts, with cortisol value ranges. All groups, EMP Aligned (*n* = 5), EMP Non-Aligned (*n* = 11), MMP Aligned (*n* = 41), and MMP Non-Aligned (*n* = 29), were concentrated in the normal cortisol range. Mean (SD) levels were 16.86 (4.20), 15.15 (5.37), 14.59 (6.00), and 15.86 (7.26) µg/dL, respectively; Table S1: Symptom characteristics on Likert scale of 1 (mild) to 5 (very severe) of the Stanford LC Cortisol Patient Cohort (*n* = 86). A value of 0 on the scale indicates symptom was not present. * Columns delineate normal, high/elevated, and low cortisol levels.
