# Supplementary material for: Can Low Cortisol Predict Long COVID? A Controversial Issue
Source: Biomedicines. 2025 Oct 27;13(11):2636. doi: 10.3390/biomedicines13112636 (PMC12650455; doi:10.3390/biomedicines13112636)
Supplement: Supplementary file 1 [file biomedicines-13-02636-s001.zip › biomedicines-3899808-supplementary.pdf]

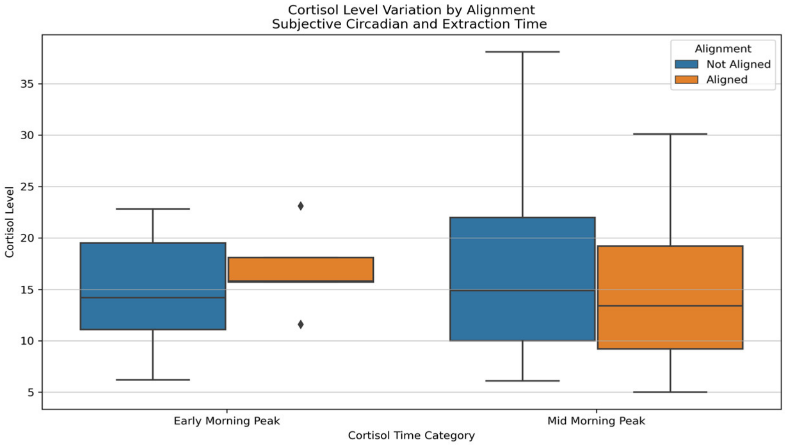

Figure S1. *Circadian and collection time alignment and non-alignment among the EMP and MMP cohorts, with cortisol value ranges. All groups, EMP Aligned (n=5), EMP Non-Aligned (n=11), MMP Aligned (n=41), and MMP Non-Aligned (n=29), were concentrated in the normal cortisol range. Mean (SD) levels were 16.86 (4.20), 15.15 (5.37), 14.59 (6.00), and 15.86 (7.26) µg/dL, respectively.*

Table S1. *Symptom characteristics on Likert Scale of 1 (mild) to 5 (very severe) of the Stanford LC Cortisol Patient Cohort (n=86). A value of 0 on the scale indicates symptom was not present. *Columns delineate normal, high/elevated, and low cortisol levels.*

Symptom	Normal*	High*	Low*	Total	*P-value*
**Anxiety and Depression**	--	--	--	--	
0 = No Symptoms	28 (51.85%)	16 (51.61%)	1 (100.0%)	45 (52.33%)	
1 = Mild	4 (7.41%)	3 (9.68%)	0 (0.0%)	7 (8.14%)	
2 = Moderate	5 (9.26%)	5 (16.13%)	0 (0.0%)	10 (11.63%)	
3 = Moderate to Severe	10 (18.52%)	1 (3.23%)	0 (0.0%)	11 (12.79%)	
4 = Severe	3 (5.56%)	5 (16.13%)	0 (0.0%)	8 (9.3%)	
5 = Very Severe	3 (5.56%)	1 (3.23%)	0 (0.0%)	4 (4.65%)	
**Brain Fog**	--	--	--	--	0.6298
0 = No Symptoms	6 (11.11%)	4 (12.9%)	0 (0.0%)	10 (11.63%)	
1 = Mild	6 (11.11%)	5 (16.13%)	0 (0.0%)	11 (12.79%)	
2 = Moderate	8 (14.81%)	1 (3.23%)	0 (0.0%)	9 (10.47%)	
3 = Moderate to Severe	8 (14.81%)	8 (25.81%)	0 (0.0%)	16 (18.6%)	
4 = Severe	14 (25.93%)	9 (29.03%)	0 (0.0%)	23 (26.74%)	
5 = Very Severe	11 (20.37%)	4 (12.9%)	1 (100.0%)	16 (18.6%)	
**Change in smell**	--	--	--	--	0.2321
0 = No Symptoms	35 (64.81%)	24 (77.42%)	0 (0.0%)	59 (68.6%)	
1 = Mild	7 (12.96%)	4 (12.9%)	1 (100.0%)	12 (13.95%)	
2 = Moderate	3 (5.56%)	0 (0.0%)	0 (0.0%)	3 (3.49%)	
3 = Moderate to Severe	2 (3.7%)	1 (3.23%)	0 (0.0%)	3 (3.49%)	
4 = Severe	3 (5.56%)	2 (6.45%)	0 (0.0%)	5 (5.81%)	
5 = Very Severe	3 (5.56%)	0 (0.0%)	0 (0.0%)	3 (3.49%)	
**Change in Taste**	--	--	--	--	0.2325
0 = No Symptoms	35 (64.81%)	24 (77.42%)	0 (0.0%)	59 (68.6%)	

1 = Mild	5 (9.26%)	4 (12.9%)	1 (100.0%)	10 (11.63%)	
2 = Moderate	5 (9.26%)	0 (0.0%)	0 (0.0%)	5 (5.81%)	
3 = Moderate to Severe	3 (5.56%)	1 (3.23%)	0 (0.0%)	4 (4.65%)	
4 = Severe	2 (3.7%)	1 (3.23%)	0 (0.0%)	3 (3.49%)	
5 = Very Severe	3 (5.56%)	1 (3.23%)	0 (0.0%)	4 (4.65%)	
**Fatigue**	--	--	--	--	0.834
0 = No Symptoms	6 (11.11%)	1 (3.23%)	0 (0.0%)	7 (8.14%)	
1 = Mild	2 (3.7%)	2 (6.45%)	0 (0.0%)	4 (4.65%)	
2 = Moderate	8 (14.81%)	2 (6.45%)	0 (0.0%)	10 (11.63%)	
3 = Moderate to Severe	11 (20.37%)	9 (29.03%)	0 (0.0%)	20 (23.26%)	
4 = Severe	7 (12.96%)	10 (32.26%)	0 (0.0%)	17 (19.77%)	
5 = Very Severe	19 (35.19%)	7 (22.58%)	1 (100.0%)	27 (31.4%)	
**Headaches**	--	--	--	--	0.596
0 = No Symptoms	15 (27.78%)	8 (25.81%)	0 (0.0%)	23 (26.74%)	
1 = Mild	6 (11.11%)	3 (9.68%)	0 (0.0%)	9 (10.47%)	
2 = Moderate	9 (16.67%)	5 (16.13%)	1 (100.0%)	15 (17.44%)	
3 = Moderate to Severe	12 (22.22%)	6 (19.35%)	0 (0.0%)	18 (20.93%)	
4 = Severe	7 (12.96%)	7 (22.58%)	0 (0.0%)	14 (16.28%)	
5 = Very Severe	4 (7.41%)	2 (6.45%)	0 (0.0%)	6 (6.98%)	
**Insomnia**	--	--	--	--	0.1233
0 = No Symptoms	16 (29.63%)	15 (48.39%)	0 (0.0%)	31 (36.05%)	
1 = Mild	4 (7.41%)	3 (9.68%)	0 (0.0%)	7 (8.14%)	
2 = Moderate	11 (20.37%)	4 (12.9%)	0 (0.0%)	15 (17.44%)	
3 = Moderate to Severe	8 (14.81%)	3 (9.68%)	0 (0.0%)	11 (12.79%)	
4 = Severe	8 (14.81%)	3 (9.68%)	0 (0.0%)	11 (12.79%)	
5 = Very Severe	6 (11.11%)	3 (9.68%)	1 (100.0%)	10 (11.63%)	
**Lethargy**	--	--	--	--	0.7443
0 = No Symptoms	14 (25.93%)	8 (25.81%)	1 (100.0%)	23 (26.74%)	
1 = Mild	2 (3.7%)	5 (16.13%)	0 (0.0%)	7 (8.14%)	
2 = Moderate	6 (11.11%)	3 (9.68%)	0 (0.0%)	9 (10.47%)	
3 = Moderate to Severe	14 (25.93%)	4 (12.9%)	0 (0.0%)	18 (20.93%)	
4 = Severe	8 (14.81%)	6 (19.35%)	0 (0.0%)	14 (16.28%)	
5 = Very Severe	9 (16.67%)	5 (16.13%)	0 (0.0%)	14 (16.28%)	
**Nasal Congestion**	--	--	--	--	0.8585
0 = No Symptoms	29 (53.7%)	17 (54.84%)	0 (0.0%)	46 (53.49%)	
1 = Mild	9 (16.67%)	4 (12.9%)	0 (0.0%)	13 (15.12%)	
2 = Moderate	8 (14.81%)	4 (12.9%)	1 (100.0%)	13 (15.12%)	
3 = Moderate to Severe	4 (7.41%)	5 (16.13%)	0 (0.0%)	9 (10.47%)	
4 = Severe	2 (3.7%)	1 (3.23%)	0 (0.0%)	3 (3.49%)	
5 = Very Severe	1 (1.85%)	0 (0.0%)	0 (0.0%)	1 (1.16%)	
**Shortness of Breath**	--	--	--	--	0.554
0 = No Symptoms	27 (50.0%)	18 (58.06%)	0 (0.0%)	45 (52.33%)	
1 = Mild	4 (7.41%)	3 (9.68%)	0 (0.0%)	7 (8.14%)	
2 = Moderate	8 (14.81%)	3 (9.68%)	0 (0.0%)	11 (12.79%)	

3 = Moderate to Severe	9 (16.67%)	4 (12.9%)	0 (0.0%)	13 (15.12%)	
4 = Severe	3 (5.56%)	1 (3.23%)	0 (0.0%)	4 (4.65%)	
5 = Very Severe	2 (3.7%)	2 (6.45%)	1 (100.0%)	5 (5.81%)	
**Unrefreshing Sleep**	--	--	--	--	0.7187
0 = No Symptoms	7 (12.96%)	2 (6.45%)	0 (0.0%)	9 (10.47%)	
1 = Mild	2 (3.7%)	2 (6.45%)	0 (0.0%)	4 (4.65%)	
2 = Moderate	10 (18.52%)	3 (9.68%)	0 (0.0%)	13 (15.12%)	
3 = Moderate to Severe	11 (20.37%)	12 (38.71%)	0 (0.0%)	23 (26.74%)	
4 = Severe	12 (22.22%)	6 (19.35%)	0 (0.0%)	18 (20.93%)	
5 = Very Severe	11 (20.37%)	6 (19.35%)	1 (100.0%)	18 (20.93%)	
